# Canal lombaire étroit par lipomatose épidurale: à propos d’un cas et revue de la littérature

**DOI:** 10.11604/pamj.2017.28.187.13233

**Published:** 2017-10-30

**Authors:** Mbaye Thioub, Maguette Mbaye, Mohamed Elhassimi Cissé, Ndaraw Ndoye, Alioune Badara Thiam, Seydou Boubakar Badiane

**Affiliations:** 1 Université Cheikh Anta Diop de Dakar, Centre Hospitalier National Universitaire de FANN, Sénégal

**Keywords:** Canal lombaire étroit, lipomatose, chirurgie, Narrow lumbar channel, lipomatosis, surgery

## Abstract

La lipomatose épidurale est une pathologie caractérisée par une l'accumulation anormale de graisse non encapsulée dans l'espace épidural. Bien que rare, elle est une cause possible de lombosciatique ou de canal lombaire étroit. Elle est souvent associée à des facteurs favorisants tels qu'une corticothérapie prolongée, ou une obésité. Nous rapportons une observation d'un patient qui a présenté des lombosciatalgies invalidantes et dont l'exploration radiologique a confirmé une lipomatose épidurale compressive. L'évolution a été favorable après décompression chirurgicale.

## Abstract (en)

Epidural lipomatosis is characterized by the abnormal accumulation of non-encapsulated grease in the epidural space. Although rare, it is a possible cause of lombosciatica or narrow lumbar channel. It is often associated with contributing factors such as prolonged corticosteroid therapy or obesity. We report the case of a patient presenting with disabling lombosciatalgias. Radiological evaluation confirmed the diagnosis of compressive epidural lipomatosis. Patient's evolution was favorable after surgical decompression.

## Introduction

La lipomatose épidurale est une affection rare caractérisée par une accumulation excessive de tissu adipeux dans l'espace épidurale. Elle est généralement secondaire à une corticothérapie locale ou générale. Il s'agit d'une cause rare de rétrécissement canalaire symptomatique. Nous rapportons le cas d'un patient pris en charge pour tableau de lombo-radiculalgie dont l'exploration radiologique a mis en évidence une étroitesse de l'espace épidural par excès de graisse épidurale.

## Patient et observation

**Figure 1:**
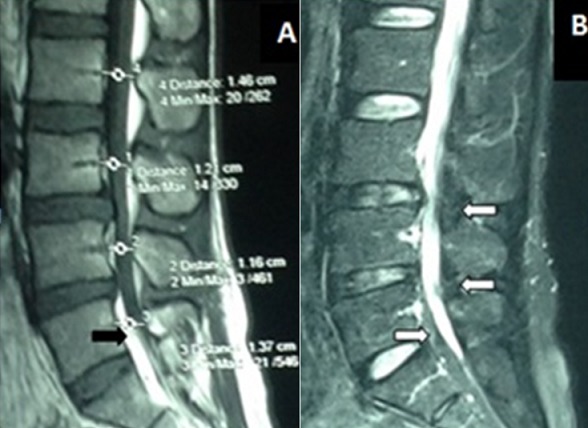
(A) IRM lombaire du patient en reconstruction sagittale la séquence T1 montre un débord discal en L3-L4 et L4-L5 associé à un épaississement compressif de la graisse épidurale antérieure hyperintense en L5 (flèche noire) et dans l’espace épidural postérieur de L2 à L5; (B) la séquence T2 montre la compression du fourreau dural par la graisse épidurale qui est hypointense en L2, L3, L4 et L5 (flèche blanche)

**Figure 2:**
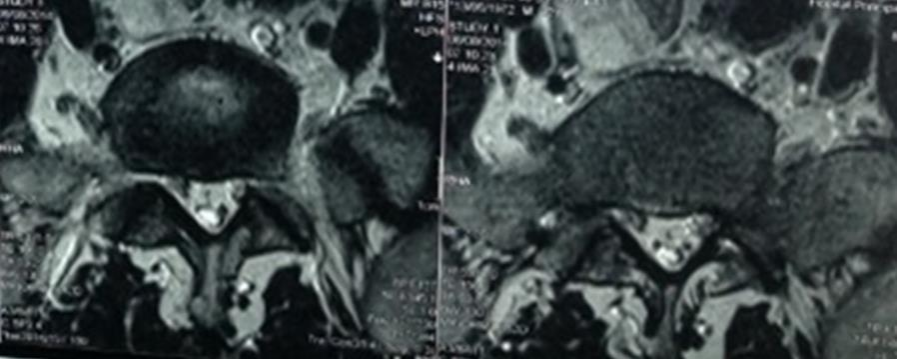
IRM lombaire du patient en coupe axiale en séquence T2; graisse épidurale en hypersignal T2 abondante avec dégénérescence graisseuse des masses musculaires para vertébrale

Un homme de 42 ans sans antécédent particulier avait consulté pour des lombalgies évoluant depuis 1 an, secondairement associées à une douleur radiculaire de type S1 gauche et de paresthésies dans le même territoire. Un traitement par anti inflammatoire non stéroïdien, antalgique de palier 2 et myorelaxant associé à la rééducation fonctionnelle pendant 2 mois, avait été institué sans succès car l'évolution a été marquée par l'installation d'une lombosciatique S1 bilatérale associée à une radiculalgie L4 gauche intermittente. Aucune prise de corticoïdes n'avait été retrouvée dans les antécédents. L'examen physique avait trouvé un bon état général, un IMC = 24.07 (78 kg pour 1m80), un Lasègue à 70° des deux côtés sans déficit neurologique. Les examens biologiques ont montré une dyslipidémie modérée: Cholestérol total = 3.37 (N = 1.5-2.4), LDL = 2.26 (N = 1-1.6), HDL = 0.92 (N = 0.3-0.6), Triglycérides = 0.91 (N<1.5). Le scanner lombaire avait trouvé des protusions discales en L3-L4 et en L4-L5 avec un canal étroit. L'IRM avait montré un canal lombaire étroit de L3 à L5 avec un débord discal en L3-L4 et L4-L5. Elle a également montré un épaississement compressif de la graisse épidurale qui apparait en hypersignal en pondération T1 de L2 à L5 en postérieur et en L5-S1 en antérieur (Figure 1, Figure 2). Une laminectomie L4 et L5 avait été indiquée. Au cours de l'intervention chirurgicale, après la laminectomie de L5, nous avons constaté quantité anormalement importante de graisse épidurale d'aspect jaunâtre et compressive sur le fourreau dural. La masse graisseuse épidurale a été complètement enlevée. A l'exploration, nous n'avons pas trouvé de protusion ou de hernie discale significative. Les suites opératoires étaient simples marquées par une régression de la lombosciatique S1 bilatérale. L'évolution à 6 mois post opératoire a été bonne avec une disparition complète de la lombo-radiculalgie et la reprise des activités.

## Discussion

La lipomatose épidurale est définie par un dépôt de graisse non encapsulée au sein de l'espace épidural rachidien. C'est une affection rare qui a été décrite pour la première fois en 1975 par Lee et al [1]. Soixante quinze pourcent des cas rapportés sont des adultes jeunes de sexe masculin [2-7]. La lombo-radiculalgie avec un caractère claudiquant associée à un examen neurologique normal est fréquemment rapportée du fait du rétrécissement canalaire. L'association de la lipomatose épidurale à une surcharge pondérale a été fréquemment rapportée et l'obésité qui est incriminée comme une des causes de cette affection, représente environ 25% des cas rapportés [2, 8]. Nous n'avons pas trouvé de surcharge pondérale chez notre patient (IMC = 24.07). Par contre nous avons noté une dyslipidémie modérée. L'augmentation des triglycérides est significativement associée à la lipomatose épidurale sans pour autant être un facteur prédictif de la probabilité de développer cette affection [9]. Les facteurs étiologiques incriminés dans la lipomatose épidurale sont: l'obésité, la corticothérapie locale ou générale et l'alcoolisme. L'administration de corticoïde est l'étiologie la mieux documentée [10]; cependant, dans certain cas la cause reste inconnue. Notre patient n'a pas reçu de corticoïdes mais il présente une dyslipidémie modérée. L'IRM chez notre patient a trouvé un canal lombaire étroit de L3 à L5 avec un débord discal compressif L3-L4 et un épaississement compressif de la graisse épidurale en L3, L4 et L5. La région thoracique est la plus touchée cependant la région lombaire est concernée dans 39 à 42% des cas; et l'étage L4-L5 est le plus touché de la région lombaire [6, 7]. Ce constat est confirmé par l'IRM de notre patient (Figure 1). Selon Chan [2], l'étroitesse canalaire ainsi que la compression de l'espace sous arachnoïdien peuvent être clairement démontrées par une IRM en coupe sagittale. Le traitement chez notre patient a été une laminectomie de L5 associée à l'exérèse totale de la masse graisseuse épidurale. Le traitement chirurgical (laminectomie associée à l'exérèse de la graisse épidurale) est l'option thérapeutique choisie par plusieurs auteurs [2, 4, 5, 11-14] dès que le patient présente des signes de souffrance neurologique. Le traitement conservateur est indiqué en première intension chez les patients obèses. Il s'agit d'un régime alimentaire aboutissant à la perte de poids. L'évolution à 6 mois post opératoire a été bonne avec une disparition complète de la lombo-radiculalgie et la reprise des activités. Ce bon résultat du traitement chirurgical a été rapporté dans la plupart des études [2, 11, 12, 15].

## Conclusion

La lipomatose épidurale est une affection rare qui entraine généralement une étroitesse canalaire chez des patients obèses ou sous corticothérapie. La pathogénicité de cette entité est reconnue par de nombreux auteurs, mais sa fréquence est probablement sous-estimée. Notre patient ne présente comme facteur étiologique qu'une dyslipidémie modérée. La laminectomie associée à l'exérèse de la graisse épidurale a donné un bon résultat.

## Conflits d’intérêts

Les auteurs ne déclarent aucun conflit d'intérêts.
